# Mechanism behind Resistance against the Organophosphate Azamethiphos in Salmon Lice (*Lepeophtheirus salmonis*)

**DOI:** 10.1371/journal.pone.0124220

**Published:** 2015-04-20

**Authors:** Kiranpreet Kaur, Kari Olli Helgesen, Marit Jørgensen Bakke, Tor Einar Horsberg

**Affiliations:** NMBU School of Veterinary Science, Sea Lice Research Centre, PO Box 8146 Dep., NO-0033 Oslo, Norway; Weizmann Institute of Science, ISRAEL

## Abstract

Acetylcholinesterase (AChE) is the primary target for organophosphates (OP). Several mutations have been reported in AChE to be associated with the reduced sensitivity against OP in various arthropods. However, to the best of our knowledge, no such reports are available for *Lepeophtheirus salmonis*. Hence, in the present study, we aimed to determine the association of AChE(s) gene(s) with resistance against OP. We screened the AChE genes (*L*. *salmonis ace1a* and *ace1b*) in two salmon lice populations: one sensitive (n=5) and the other resistant (n=5) for azamethiphos, a commonly used OP in salmon farming. The screening led to the identification of a missense mutation *Phe362Tyr* in *L*. *salmonis ace1a*, (corresponding to *Phe331* in *Torpedo californica* AChE) in all the samples of the resistant population. We confirmed the potential role of the mutation, with reduced sensitivity against azamethiphos in *L*. *salmonis*, by screening for *Phe362Tyr* in 2 sensitive and 5 resistant strains. The significantly higher frequency of the mutant allele (*362Tyr*) in the resistant strains clearly indicated the possible association of *Phe362Tyr *mutation in *L*. *salmonis ace1a* with resistance towards azamethiphos. The 3D modelling, short term survival experiments and enzymatic assays further supported the imperative role of *Phe362Tyr *in reduced sensitivity of *L*. *salmonis* for azamethiphos. Based on all these observations, the present study, for the first time, presents the mechanism of resistance in *L*. *salmonis *against azamethiphos. In addition, we developed a rapid diagnostic tool for the high throughput screening of *Phe362Tyr* mutation using High Resolution Melt analysis.

## Introduction

Acetylcholinesterase (AChE), encoded by *ace* genes, is a serine hydrolase that plays a critical role in neurotransmission at cholinergic synapses and neuromuscular junctions. AChE is a target for two main classes of anti-cholinergic agents, organophosphates (OP) and carbamates (CB). OP and CB bind to the active site of AChE, and inactivate the enzyme by phosphorylating or carbamylating a serine residue in the enzyme’s catalytic center [1]. The binding blocks the cleavage of the transmitter, acetyl choline (ACh), and results in elevated levels of ACh in the synaptic cleft thereby causing excitation, paralysis and death [2].

OPs have been used for treatment against salmon lice (*Lepeophtheirus salmonis*), a marine ectoparasitic copepod on salmonid species, in Norwegian salmonid aquaculture since the late 1970s. The first agent used was metrifonate (Neguvon), followed by dichlorvos (Nuvan) in 1986 and azamethiphos (Salmosan) in 1994 [3]. In 1991, the first cases of reduced efficacy of organophosphate treatments were noted in Mid-Norway [4]. When the use of azamethiphos was terminated during 1999, the problem of reduced sensitivity in salmon lice against azamethiphos was wide-spread. At that time, the cause of resistance was not determined.

Azamethiphos was re-introduced as a treatment agent against salmon lice in 2008 [5]. We received new reports of reduced efficacy of treatments with azamethiphos from the field in 2009. In 2013, a surveillance program, using bioassays to test for resistance, revealed a widespread distribution of azamethiphos resistance in Norwegian fish farms [5]. Bioassays are toxicological tests performed on live parasites and are thus labor intensive and associated with several sources of biases. Understanding the biochemical pathways underlying resistance in *L*. *salmonis* would therefore lead to the development of better tools to determine and control resistance. This would possibly improve management strategies and help in preventing economical loss due to ineffective treatments in the aquaculture industry.

Known resistance mechanisms towards organophosphates in arthropods include behavioral factors (the arthropod avoids the agent) and metabolic factors (e.g. enhanced activity of glutathion S-transferase or unspecific esterases) [6]. However, point mutations in AChE have been reported to be the most common mechanism behind reduced sensitivity in arthropods against OP [7].

Unfortunately, to the best of our knowledge, no study is available in the recent literature on AChE as a target site of OP in *L*. *salmonis*. We have recently identified and characterized the two genes coding for AChE in *L*. *salmonis* [8]. The full length cDNA sequences encoding the two AChEs in *L*. *salmonis* were identified and fully characterized. Complete cDNA sequence encoding the *L*. *salmonis ace1a* (GenBank KJ132368) and *ace1b* (GenBank KJ132369) and the deduced amino acid sequences were determined. The two AChEs were highly similar to each other (84% similarity at protein level), an observation quite unique to *L*. *salmonis* and has not been observed in other arthropods previously. *Ace1a* was predominantly expressed in different developmental stages of salmon lice compared to *ace1b* and was active in the cephalothorax, indicating that *ace1a* plays the major role in synaptic transmission [8].

In the present study, we aimed to determine the cause of reduced sensitivity in salmon lice against azamethiphos. This was achieved by screening the two acetylcholinesterase genes (*ace1a* and *ace1b*), in both sensitive and resistant *L*. *salmonis* populations. In addition, the effect of changes identified, on the expression, protein structure, activity of AChE and finally the survival of *L*. *salmonis* was also investigated and accomplished.

## Materials and Methods

### Salmon lice strains and phenotypic characterization

Salmon lice samples were collected in the field. Four strains were kept in continuous culture [9] in the laboratory at The Norwegian Institute for Water Research’s Marine Research Station at Solbergstrand, Drøbak (NIVA) or at the Institute of Biology, University of Bergen (UiB). The fish were anesthetized for handling procedures using Finquel vet (tricain mesilat, Western Chemical Inc., USA) dissolved in fresh water at final concentration of 125 mgL^-1^ sea water. The fish were sacrificed in an anesthesia bath containing an overdose of the same substance. The Atlantic salmon applied as parasitic hosts at NIVA came from the commercial supplier Sørsmolt in Kragerø, Norway, while the rainbow trout came from the Norwegian University of Life Sciences (UMB) at Ås, Norway. The Atlantic salmon at UiB came from the breeding station of the Institute of Marine Research at Matre, Norway.

To characterize the salmon lice strains with regard to their sensitivity to azamethiphos, small scale treatments of fish infested with salmon lice were performed. In addition the sensitivity was tested by performing biological assays (bioassays) on salmon lice detached from the fish. The different strains of salmon lice included in the current study (Ls A, Ls G, Ls B, Ls H, Ls H-s, Ls V, Ls F and Ls 1998) are presented in Table 1 with their treatment history prior to collection, whether small scale treatments have been performed, and which type of bioassays have been performed.

**Table 1:** History and phenotypic classification of salmon lice samples included in the study.

Strain	History	Lice bioassay	Small scale fish treatment
Ls A	Sampled in 2010, cultivated for 10 generations, never treated with azamethiphos	60 min., 24 h.	+
Ls G	Sampled in 2004, cultivated for 15 generations, not treated with azamethiphos for 8 years prior to sampling	60 min., 24 h.	_
Ls B	Sampled in 2008, site treated 3 times with azamethiphos the last two years prior to sampling	60 min.	_
Ls H	Sampled in 2011, site treated with azamethiphos more than 5 times for the last two years	60 min., 24 h.	+
Ls H-s	Surviving parasites after a small-scale lab-treatment of Ls H with azamethiphos	_	_
Ls V	Sampled in 2012 immediately after an azamethiphos treatment. The site had been treated with azamethiphos more than 5 times for the last two years	60 min.*	_
Ls F	Sampled in 2013, site treated with azamethiphos more than 5 times for the last two years	24 h.	+**
Ls 1998	Surviving parasites after an azamethiphos bioassay selection experiment in 1998, stored at -80°C until analysis	_	_

* Tested at one concentration only

** Performed to identify when the different genotypes detached

The eight salmon lice strains included in the current study are presented with their treatment history with azamethiphos prior to parasite collection (Ls A, Ls G, Ls B, Ls H, Ls V and Ls F) or in the laboratory (Ls H-s and Ls 1998). The salmon lice strains were exposed to azamethiphos for 60 minutes and/or 24 hours in biological assays (bioassays) to detect their sensitivity to the chemical. This information is stated in the table. Whether or not salmon infested with salmon lice from the different strains were subjected to small scale treatment trials to detect treatment efficacies are also given in the table. The small scale treatment of salmon infested with Ls F was performed to detect which genotypes of the parasite that died at different time points during and after the treatment.

The small scale treatments for efficacy evaluation were performed by treating one group of Atlantic salmon or rainbow trout infested with preadult parasites, of the salmon lice strain to be tested, with Salmosan (50% w/w azamethiphos, Fish Vet Group, UK) at a concentration of 0.1 mgL^-1^ azamethiphos and keeping a separate group as untreated controls. The water exchange in the tanks was stopped for 30 minutes before the solution was drained and the tank rapidly refilled. The water was oxygenated during the treatment. The treatment effect was evaluated 5–7 days post treatment by counting parasites in both treatment and control groups. The first small scale treatment of Ls A and the small scale treatment of Ls H was performed at NIVA on rainbow trout and the results have been reported previously [10]. The second treatment of fish infested with Ls A was performed at UiB treating 6 Atlantic salmon weighing 300 grams each and keeping 6 fish as untreated control in another 500 liter tank. The fish had initially been infested with salmon lice in a common infestation trial. The effect of the treatment was evaluated 5 days post treatment.

The small scale treatment for genotype identification was performed on Ls F to see what genotypes that died at different time intervals during and after treatment. It was performed at NIVA on three salmon weighing 100 grams each which were infested with Ls F. The treatment was performed in a 100 liter tank, the same way as the other small scale treatments, but without a control group. During the exposure period and the first 2.5 hours thereafter, all sea lice detached from the fish were picked out of the tank and put on RNA-later. 24 hours post exposure all detached lice were removed from the tank and put on RNA-later. Seven days later all remaining sea lice were removed from the fish and put on RNA-later. Three parasites had detached from the fish, but were alive and attached to the wall of the tank at 2 hours after initiation of the treatment. These were excluded from the analysis. After the experiments the fish were sacrificed in a lethal anaesthesia bath.

The sensitivities of the lab-cultivated strains towards azamethiphos were characterized by two types of bioassays and small scale treatments.

The 60 minute bioassay was performed on Ls A, Ls G, Ls B, Ls H and Ls V as described by Helgesen and Horsberg [10]. The results from Ls A and Ls H have been remodeled from data presented in Helgesen and Horsberg [10]. Preadult parasites were exposed to six different concentrations of azamethiphos (0–140 μgL^-1^, different concentrations in different assays) in polystyrene boxes. After 60 minutes exposure, the boxes containing the parasites were rinsed in fresh sea water and kept in clean, aerated sea water at 12°C for 24 hours before the parasites were characterized as either alive or dead/immobilized [10].

The 24 hour bioassays were performed on Ls A, Ls G, Ls H and Ls F by exposing preadult parasites to six different concentrations of azamethiphos (0–2 μgL^-1^, different concentrations in different assays) in sea water for 24 hours, using glass bottles kept at 12°C with constant aeration [10]. The results were read after 24 hours exposure by gently turning the bottles and thereafter pouring the solution into a beaker. Parasites attached to the bottle wall as well as parasites able to attach to the beaker wall or swim in a straight line, were characterized as alive. All other parasites in the beaker were categorized as dead/immobilized. The results from Ls A and Ls H has previously been presented in Helgesen and Horsberg [10].

Frozen samples (both surviving and immobilized parasites, n = 9) from a bioassay performed in 1998 (a not given concentration of azamethiphos) were also enrolled in the study.

### Total RNA extraction and cDNA synthesis

Total RNA was extracted using RNeasy plus Mini kit (Qiagen, CA, USA), from female adult individuals, as per manufacturer’s protocol. The RNA was quantified and qualified on ND-100 Spectrophotometer (Thermo Fisher Scientific, DE, USA). First strand cDNA was synthesized from total RNA (1μg) using qScript reverse transcriptase (Quanta Biosciences, MD, USA).

### Screening of full length *L*. *salmonis ace1a* and *ace1b*

Full length cDNAs (*ace1a* and *ace1b*), from 5 sensitive (Ls A) and 5 resistant (Ls H) adult female sea lice samples, were amplified using gene specific primers (mentioned below). PCR reactions were performed using Phusion high-fidelity DNA polymerase (New England BioLabs, MA, USA) under the conditions: 98°C for 30 s, followed by 35 cycles at 98°C for 10 s, 55°C for 15 s, 72°C for 2 min followed by a final extension at 72°C for 10 min. Amplicons were then subjected to direct sequencing using BIG Dye Terminator v3.1 cycle sequencing kit (Life technologies, Invitrogen, CA, USA) on 3130xl Genetic Analyzer (ABI Prism, Life technologies, Invitrogen, CA, USA).

Primers used to amplify the whole cDNA

*ace1a* forward primer: CTCTGCTGCTACACCGACTCCTGTT

*ace1a* reverse primer: TCGAGGATGTTTGACACTGATGGTC

*ace1b* forward primer: TGTTTTAGATGTGGATTCAAGTCCGAA

*ace1b* reverse primer: CGATGGATGGTACGTACGTATGAACATA

### Screening of missense changes identified in *L*. *salmonis* a*ce1a* and *ace1b*

50 adult female samples each from 2 sensitive (Ls A and Ls G) and 2 resistant populations (Ls H, Ls B) were screened by direct sequencing. In addition, 2 *L*. *salmonis* populations (24 samples of Ls H-s and 20 samples of Ls V) that survived the azamethiphos treatment were also screened for these missense changes by direct sequencing.

Primers to amplify missense change in *L*. *salmonis ace1a*

*ace1a* forward primer: GTGGATGGAAGTTTCTTGGATGAGAG

*ace1a* reverse primer: CTCAAAAGTTATTGCCTCTCTTCCCATT

Primers to amplify missense change in *ace1b*

*ace1b* forward primer: ACGAGCAAAGTCAGCAGTTG

*ace1b* reverse primer: TTTCATCCGCAGTGTTTCAG

### Genotyping

The *Phe362Tyr* mutation in *L*. *salmonis ace1a*, which corresponds to codon 331 in the *Torpedo californica* AChE, was validated by High Resolution Melt (HRM) analyses, which is a simple rapid tool to screen single base changes (mutations/polymorphisms) with high sensitivity and accuracy [11]. The methodology included the generation of specific PCR product using gene specific primers (mentioned below) and Precision Melt supermix (Bio Rad, CA, USA), as per manufacturer’s instructions, with a sensitive fluorescent dye (EvaGreen) that binds specifically only to double stranded DNA, followed by subjecting the amplicon to gradual increase in temperature (65°C to 95°C), which led to the denaturation of double stranded amplicon and decrease in fluorescence. This change in florescence was recorded by the C1000 Touch thermal cycler (Bio-Rad, CA, USA) as a melt curve (fluorescence versus temperature). The samples were assembled into different groups based on difference in the shapes of their melt curves.

HRM primers for the *Phe362Tyr* mutation

*Forward primer*: TTTTAATTGGAGCGAATAAGGA

*Reverse Primer*: TCTGTTCGATCAACATAGACG

The typing of parasites from the small scale treatment for genotype identification were performed by qPCR using TaqMan probes specific for the sensitive (S) and resistant (R) genotypes. The assay was developed for high throughput analyses by PatoGen Analyse AS, based on the results presented here. By combining the probes, each parasite could be classified as sensitive (SS), heterozygote (RS) or homozygote resistant (RR). Genotyping could be performed in all except one parasite that was dead at 24 hours after initiation of the treatment.

### Alignment of amino acid sequences

Deduced amino acid sequences of *L*. *salmonis* AChE1a and AChE1b were compared with 33 previously published AChE protein sequences from other species, using CLUSTALW program with BLOSUM matrix and default settings [12] to obtain Multiple sequence alignment (MSA).

### 3D modelling of the enzymes

The three-dimensional structure of the AChE1a enzyme from *L*. *salmonis* was modeled using SWISS MODEL in the automated mode [13] (http://swissmodel.expasy.org/). An initial template search using the wild-type AChE1a from *L*. *salmonis* as target revealed several possible templates. The best fit was found with native AChE from *D*. *melanogaster*, PDB-ID 1qo9 [14] (RMS 0.25 for the whole protein, 0.05 for ten amino acids important for choline binding, the catalytic triad, the acyl pocket and the oxyanion hole). The Root Mean Square (RMS) for the fit between template and target were calculated using the Swiss PDB viewer 4.1.0. (http://www.expasy.org/spdbv/). Azamethiphos was docked to the wild-type and the mutated AChE1a using the online molecular docking server (http://www.dockingserver.com/web) and the best fit was illustrated using the UCSF Chimera 1.10.1. software (http://www.cgl.ucsf.edu/chimera/).

### Inhibition of enzymatic activity

Two approaches were used to assess inhibition of AChE activity and possible differences between OP-susceptible and-resistant *L*. *salmonis* strains. In both the experiments, only preadult females were used. Lice from the susceptible strain (Ls A) were all expected to be SS. This was based on the frequency of the genotype SS (100%) in the screening of this strain. All the samples from the resistant strain were individually cut in two with a sterile scalpel. One half was put in RNA *later* for subsequent genotyping, whereas its counterpart was stored at -80°C for enzymatic assay. This allowed the use of only confirmed RR-lice in the assays.

### *In vitro* treatment

To assess the importance of the *Phe362Tyr* mutation (corresponds to Phe331 in *Torpedo californica* AChE) *in vitro*, a slightly modified version of a protocol developed by the World Health Organization (WHO) to detect insecticide resistance mechanisms in mosquitoes (WHO/CDS/CPC/MAL/98.6) was used [15]. The modifications were needed to optimize the protocol for sea lice. In brief, samples (one whole or two half preadult II/ two whole or four half preadult I) were homogenized in 75 μl deionized (18 MΩ) water with a pestle. To reduce the influence of protease activity, the samples were prepared just a few minutes before the assay was started and were kept on ice at all times. The enzymatic activity was analyzed using the principle of Ellman *et al*. (1961) on 96 wells microtitre plates [16]. The wells contained phosphate buffer (0.1 M, pH 7.8) with 1% Triton X-100 (140 μl), 5,5′-Dithiobis-(2-nitrobenzoic acid) in phosphate buffer (10 mM work solution; 10 μl) and 10 mM acetylthiocholine iodide (ATC) in deionized water (10 mM work solution; 25 μl). In a parallel series, propoxur (0.1 M in acetone) was added to the ATC work solution giving a concentration of 0.2 mM propoxur. The ingredients in the wells were gently mixed before the addition of lice homogenate (25 μl) to both parallels. This allowed for comparison of AChE activity with and without propoxur inhibition (n = 21 for RR and n = 20 for SS). The microtitre plates were shaken for 1 minute and immediately read in kinetic mode for 10 minutes at 405 nm (Epoch spectrophotometer, BioTek, USA). The individual slopes were calculated based on the best linear fit (Gen5 version 2.00) and a standard curve prepared from AChE from electric eel (SigmaAldrich) was used to calculate the enzymatic activity. One unit (U) is the amount of enzyme expected to catalyze 1 μmole substrate per minute. For each sample the activity is expressed relative to the protein content in the homogenate (Umg^-1^ protein). Protein content was measured on a Take3 plate in an Epoch spectrophotometer (BioTek Instruments Inc., USA) and calculated in Gen5 version 2.0 using a build-in standard curve for bovine serum albumin (BioTek Instruments Inc., USA). Inhibition in percent of normal AChE activity was calculated for each sample (= 100-(activity with propoxur*100)/ activity without propoxur).

### *In vivo* treatment

Preadult female lice were collected alive and randomly assigned to either a 0 μgL^-1^ or 2 μgL^-1^ azamethiphos bath exposure for 24 hours. The exposures were carried out on the detached parasites in filtered and continuously aerated sea water in 1 liter glass bottles kept at 10°C. Susceptible and resistant strains were kept in separate bottles. After 24 hours the lice were collected and sampled as described above. Residual AChE activity was measured following the modified WHO protocol (see *in vitro* section). Because azamethiphos was used to block AChE activity, no propoxur was added to the mixtures. AChE activity could not be measured before and after treatment in the same individual, hence the results are presented as absolute values instead of relative values.

### Statistics

All bioassay results were modelled using probit analysis in the statistical software JMP 10 (SAS Institute Inc., Cary, NC, USA) and EC_50_-values (the concentration immobilizing 50% of the parasites) with 95 percent confidential intervals were calculated. The mean treatment efficacies in the small scale treatments were calculated using bootstrapping with 2500 simulations calculating the difference (in %) of parasites between the treated groups and their respective control groups using JMP. The 95% confidence intervals (CI) for efficacy were constructed using the number of fish per treated group as N.

An analysis of survival versus time for the three genotypes was then conducted with a Kaplan-Meier survival analysis including a Wilcoxon test (JMP).

The results from the enzymatic inhibition studies were statistically compared (JMP) with ANOVA after root transformation (*in vitro* study) or the non-parametric multiple comparison Steel-Dwass method (*in vivo* study).

### Ethics Statement

The studies were approved by the NIVA local ethics committee, ID 2995, in accordance with the guidelines set by The Norwegian Animal Research Authority. The research station is approved as a fish research facility by the Norwegian Animal Research Authority.

## Results

### Bioassays and small scale treatment for phenotypic characterization

All results from small scale treatments and bioassays, performed to characterize the salmon lice strains with regard to their sensitivity to azamethiphos, are given in Table 2. Ls A showed high mortalities in the two small scale treatments, 100% and 98%, respectively. Both Ls A and Ls G showed low EC_50_-values (< 3 μgL^-1^ in the 60-minutes and < 0.2 μgL^-1^ in the 24-hour bioassays). Fifty percent of the sea lice from Ls H died in the small scale treatment. The EC_50_-values (60-minutes assay) from this strain was more than 28 times higher than the values from Ls A and Ls G. The EC_50_-value (60-minutes assay) from Ls B was higher than the corresponding value in the 60-minutes assay on Ls A but much lower than the Ls H EC_50_-values from the 60-minutes bioassay. The strain used in the small scale treatment experiment for genotype identification, Ls F, demonstrated sensitivity that was lower than Ls A and Ls H in a 24-hour bioassay.

**Table 2:** The results from bioassays with 60 minutes and 24 hours exposure to azamethiphos.

Sea lice strain	EC_50_, 60-min bioassay	EC_50_, 24-h bioassay	Percent efficacy, small scale treatment	Classification
Ls A	2.1 (1.3–3.5)*	0.12 (0.11–0.14)*	100 (90–100)*, 98 (82–100)	Sensitive
Ls G	1.8 (1.4–2.5)	0.16 (0.10–0.27)		Sensitive
Ls B	4.5 (1.9–10.7)	_	_	Reduced sensitivity
Ls H	60 (17–216)*	2.1 (1.5–2.7)*	50 (39–61)*	Resistant
Ls V	>50**	_	_	Resistant
Ls F	_	3.3 (1.9–5.6)	_	Resistant

*Remodeled from data presented in Helgesen and Horsberg [10]

**Tested at one concentration only

The results from bioassays with 60 minutes and 24 hours exposure to azamethiphos are given as EC_50_-values (the concentration that immobilizes 50% of the parasites) in μgL^-1^ with 95% confidence intervals (CI). The bioassay results were modelled using probit modelling in JMP 10 (SAS Institute Inc., Cary, NC, USA). The results from the small scale treatments of salmon infested with Ls A and Ls H in a 30 minute bath treatment with 0.1 mgL^-1^ azamethiphos and an untreated control group The results from the small scale treatments were calculated using bootstrapping and are given as percent effect with 95% CI.

### Screening of the *L*. *salmonis ace1a* and *ace1b* genes for polymorphisms

**Fig 1:**
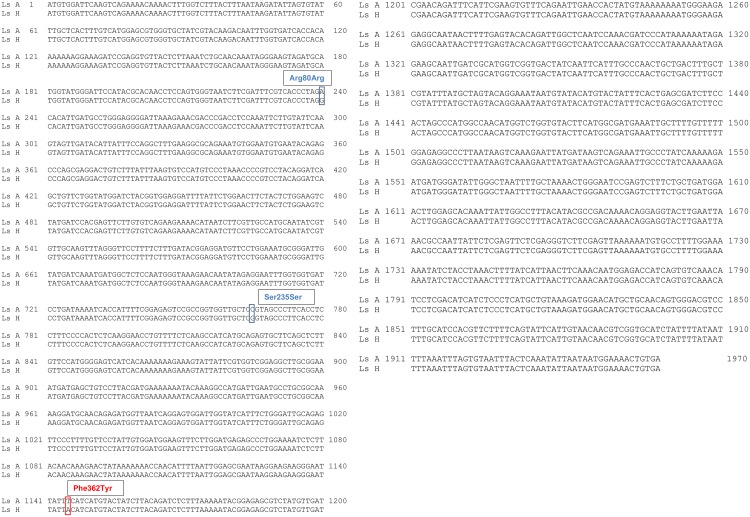
Nucleotide alignment of *ace1a*. Nucleotide alignment of *L*. *salmonis ace1a* from sensitive (Ls A) and resistant (Ls H) salmon lice strains. The changes identified are boxed. Of the three nucleotide changes identified, two were silent changes, *Arg80Arg* and *Ser235Ser*, corresponding to *Glu49* and *Ser176* in *T*. *californica* AChE, respectively. The non-silent T->A change led to the substitution of Phe to Tyr residue at 362 amino acid position corresponding to *Phe331* in *T*. *californica* AChE.

The screening of whole cDNA sequence of both the genes in five sensitive (Ls A) and five resistant (Ls H) salmon lice revealed one non-synonymous change and two silent changes in *L*. *salmonis ace1a*. The non-synonymous change led to an amino acid change: phenylalanine to tyrosine at codon 362, which corresponds to codon 331 in the *Torpedo californica* amino acid sequence. The two other substitutions were silent changes (Fig 1).

**Fig 2:**
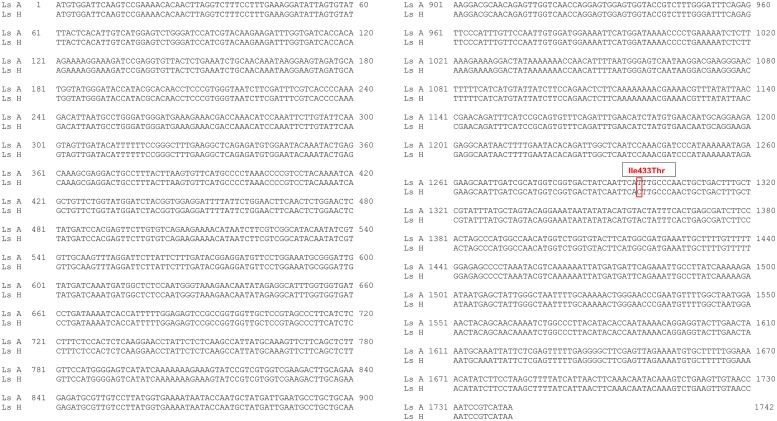
Nucleotide alignment of *ace1b*. Nucleotide alignment of *L*. *salmonis ace1b* from sensitive (Ls A) and resistant (Ls H) salmon lice strains. The only change identified in *L*. *salmonis ace1b* is boxed. This non silent T->C change led to the substitution of Ile to Thr at 433 amino acid position, corresponding to *Ile401* in the *T*. *californica* AChE.

In *ace1b*, a single change was identified in codon 433, leading to Isoleucine-> Threonine substitution (Fig 2), which corresponds to codon Ile401 in the *T*. *californica* amino acid sequence. The frequencies of this change is listed in the supporting information, S1 Table.

### Association of missense changes in *ace1a* and *ace1b* with resistance against azamethiphos

Both the non-synonymous changes (*Phe362Tyr* in *ace1a* and *Ile433Thr* in *ace1b*) were screened, by direct sequencing, in laboratory cultured sea lice populations, including the two sensitive strains (Ls A, Ls G) and the two strains with reduced sensitivity (Ls B, Ls H) to determine their association with resistance against azamethiphos. Fifty samples from each population were enrolled for screening. None of these populations were under any treatment pressure when enrolled. In addition, 20 parasites that survived a normal field treatment with azamethiphos (Ls V) along with 24 samples from Ls H surviving a small scale azamethiphos treatment were also screened for the *Phe362Tyr* change. The results (Table 3) demonstrated a clear association between the sensitivity classification and the frequency of the *Phe362Tyr* mutation. However, no such association was observed for the *Ile433Thr* change in *L*. *salmonis ace1b* and the sensitivity classification (S1 Table).

**Table 3:** Frequency of the Phe->Tyr change in codon 362 of *ace1a* (*L*. *salmonis*), corresponding to codon 331 in *T*. *californica*.

Strain	Sensitivity	Wild type Phe362/Phe362 frequency (SS)	Heterozygote Phe362/362Tyr frequency (RS)	Homozygote 362Tyr/362Tyr frequency (RR)
Ls A	Sensitive	100%	0%	0%
Ls G	Sensitive	96%	4%	0%
Ls B	Reduced sensitivity	72%	26%	2%
Ls H	Resistant	44%	36%	20%
Ls H-s (azamethiphos treatment)	Resistant	0%	92%	8%
Ls V	Resistant	5%	35%	60%

### *Phe362Tyr* in samples collected in 1998

The salmon lice samples (n = 9) after the selection experiment with azamethiphos in 1998 were also screened for the *Phe362Tyr* change. The screening revealed that this change was present in all the salmon lice (n = 4) that survived the exposure (SS = 0, RS = 3 and RR = 1) at that time. None of the samples that died during azamethiphos exposure harbored the mutation (n = 5).

### Phe362Tyr

**Fig 3:**
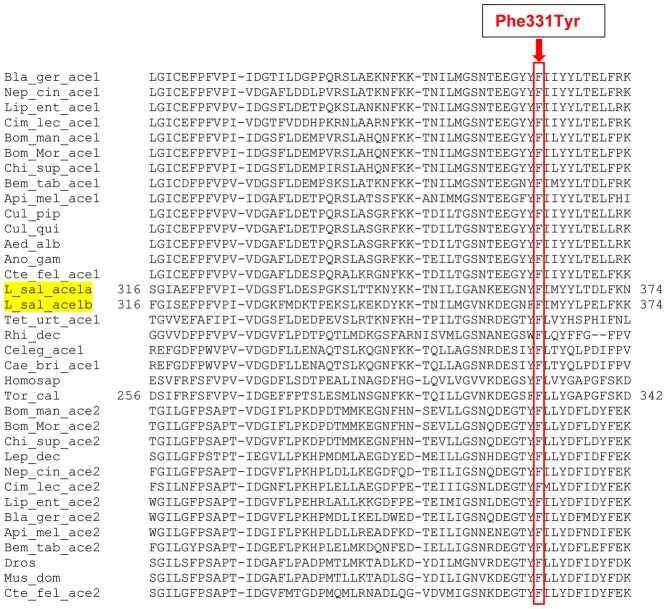
Amino acid alignment. Alignment of the deduced amino acid sequence of both *L*. *salmonis ace1a* and *ace1b* in the region where the *Phe362Tyr* change was found, with previously published acetylcholinesterases (AChE) from other insects, arachnida and vertebrates (Insects: *Liposcelis entomophila* Lip_ent, *Bemisia tabaci* Bem_tab, *Blattella germanica* Bla_ger, *Nephotettix cincticeps* Nep_cin, *Ctenocephalides felis* Cte_fel, *Culex pipiens* Cul_pip, *Chilo suppressalis* Chi_sup, *Apis mellifera* Api_mel, *Cimex lectularius* Cim_lec, *Bombyx mandarina* Bom_man, *Bombyx mori* Bom_Mor, *Leptinotarsa decemlineata* Lep_dec, *Drosophila melanogaster* Dros, *Musca domestica* Mus_dom, *Anopheles gambiae* Ano_gam, *Aedes albopictus* Aed_alb, *Culex quinquefasciatus* Cul_qui, Arachnida: *Tetranychus urticae* Tet_urt, *Rhipicephalus decoloratus* Rhi_dec, Vertebrates: *Torpedo californica* Tor_cal, *Homo sapiens* Homosap. *Phe362Tyr* corresponds to *Phe331* in *Torpedo californica* AChE. Phenylalanine at 331 is a highly conserved amino acid in the acetylcholinesterases among all species included. The *Phe331Tyr* change is boxed.

The alignment of the *L*. *salmonis* AChE1a protein with 33 AChE amino acid sequences from other species revealed that the *Phe362Tyr* in *ace1a* is homologous to *Phe331* of AChE in *T*.*californica* and is located in the acyl pocket neighboring the catalytic center in the active site gorge. It is a highly conserved residue among the species as evident from multiple sequence alignment (MSA) of AChEs from different species (Fig 3).

### 3D modelling of the enzyme

The 3D modeling was performed using SWISS MODEL http://swissmodel.expasy.org/ [13], http://swissmodel.expasy.org/. The 3D structure of the native enzyme from *Drosophila melanogaster* (PDB ID: 1qo9) was used as a template. The protein from *L*. *salmonis* could fit the template, but the fit was not optimal. The QMEAN4 score (a parameter between 0 and 1 where a higher number indicates a better fit) was 0.541. However, the Root Mean Square (RMS) values were low, 0.25 for the whole protein and 0.05 for ten essential amino acids. Thus, the models were still considered useful.

The generated pdb files are included in the supplementary material (S1 File and S2 File).

**Fig 4:**
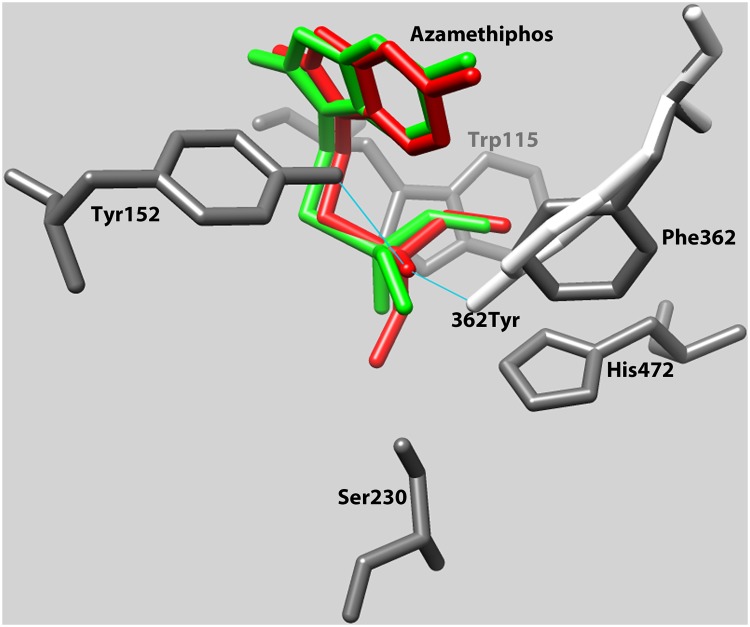
3D model of important amino acids. Overlay of the predicted three-dimensional positioning of functionally important amino acids in AChE1a wild-type and mutated enzyme from *Lepeophtheirus salmonis*. The changed amino acid (*362Tyr*, corresponding to codon 331 in *T*. *californica*) is displayed in white. The other amino acids, *Trp115*, *Tyr152*, *Ser230* and *His472* (*Trp84*, *Tyr130*, *Ser200* and *His440* in *T*. *californica*) are displayed in grey. Other amino acids are not displayed. The *Phe362Tyr* mutation alters the structure and the polarity of the enzymatic pocket. According to the ligand docking model, azamethiphos (green in the wild-type AChE1a, red in the mutated AChE1a) binds differently in the pocket, with H-bonds to both *Tyr152* and *362Tyr* in the mutated enzyme. No H-bonds were predicted between azamethiphos and these two amino acids in the wild-type enzyme. SWISS MODEL in the automated mode [13] (http://swissmodel.expasy.org/) was used for modelling of the protein, the molecular docking server (http://www.dockingserver.com/web) was used to dock azamethiphos to the protein, and Chimera 1.10.1. (http://www.cgl.ucsf.edu/chimera/) was used to illustrate the positions.

The 3D model (Fig 4) revealed that the change to *Tyr* at position *362*, resulted in interference with the entrance to the catalytic triad of the enzyme (*Ser230*, *Glu358* and *His472* in *L*. *salmonis*, corresponding to *Ser200*, *Glu327*and *His440* in *T*. *californica*). The aromatic ring of *Tyr* is turned approximately 50 degrees compared to the aromatic ring of *Phe*. Tyrosine has a hydroxyl group in the para-position, which enters the groove leading to the catalytic triad, decreasing the volume of the pocket. The substitution of the nonpolar *Phe* with the polar *Tyr* also changes the polarity of the active gorge and thereby the binding site for organophosphates, most likely affecting binding of these molecules in the enzyme. The best fit of azamethiphos in the catalytic gorge of the wild-type and in the mutated enzyme implied hydrogen (H) bonds between *362Tyr* and azamethiphos, and between *Tyr152* and azamethiphos. The model did not predict H-bonds between *Phe362* and azamethiphos, or between *Tyr152* and azamethiphos in the wild-type enzyme.

### High Resolution Melt analysis (HRM)

**Fig 5:**
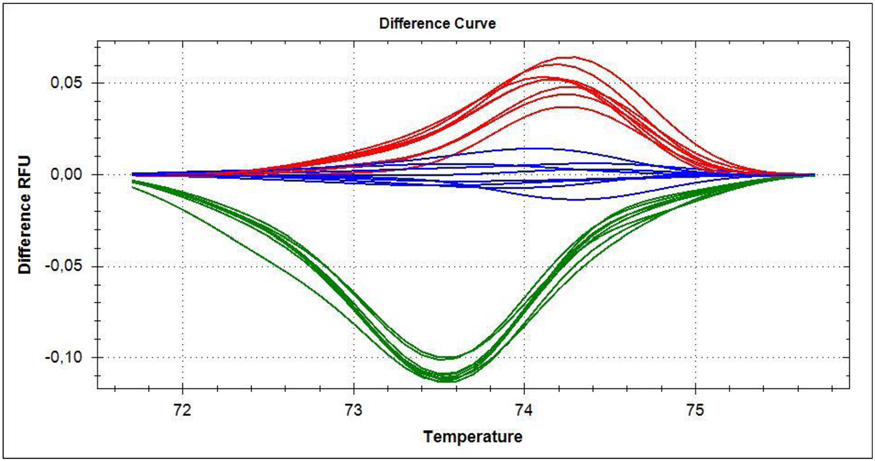
High resolution melt plots. High Resolution Melt (HRM) Analysis separated the samples with and without the *Phe362Tyr* mutation (numbering from *L*. *salmonis*). HRM was based on differences in the shapes of their melt curve that reflects the differences in their genotypes. The Green cluster represents samples homozygous (RR) for the *Phe362Tyr* mutation, Blue cluster represents samples heterozygous (RS) for the *Phe362Tyr* mutation and Red cluster represents the wild type (SS) samples without the *Phe362Tyr* mutation, respectively. All the three clusters were clearly separated from each other on the HRM plot.

High Resolution Melt analysis (HRM) was performed to validate the sequencing results and in an attempt to develop a rapid diagnostic tool for the detection of *Phe362Tyr* mutation in *L*. *salmonis ace1a*. After standardizing the technique with samples of known genotypes, determined by direct sequencing (wild type, heterozygous and homozygous for *Phe362Tyr* mutation), samples with unknown genotypes were run to confirm the results obtained. These were also confirmed by direct sequencing. HRM analysis could distinguish between the samples of different genotypes with high accuracy. As shown in Fig 5, the samples were very well separated based on their genotypes.

### Treatment trial for genetic characterization

The frequency of live and dead parasites from Ls F within each genotype is given in Table 4. All parasites of the SS genotype died within two hours after initiation of the treatment, while no parasites of the RR genotype died within eight days after the treatment. The mortality rate of the RS genotype was 44%.

**Table 4:** Mortality frequency of homozygote sensitive (SS), heterozygote (RS) and homozygote resistant (RR) parasites over eight days following a 30 minute bath treatment with 0.1 mgL^-1^ azamethiphos.

Genotype	Dead	Alive
SS	10	0
RS	11	14
RR	0	16

Mortality frequency of homozygote sensitive (SS), heterozygote (RS) and homozygote resistant (RR) parasites over eight days following a 30 minute bath treatment with 0.1 mgL^-1^ azamethiphos. Three salmon infested with Ls F were treated and all detached salmon lice were removed during the exposure and the following 2.5 hours. Detached parasites were also removed 24 hours later. They are presented in the “dead” column except two parasites which were excluded as they attached to the tank wall after detaching from the fish. The rest of the parasites were picked off the fish 8 days post treatment and these are presented in the “alive” column. All salmon lice were genotyped by PatoGen AS in Ålesund, Norway using a TaqMan assay.

**Fig 6:**
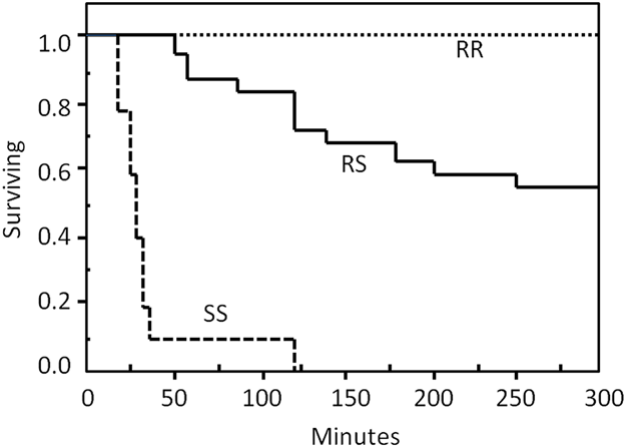
Survival analysis plot. Kaplan-Meier survival plot of all three genotypes: homozygote sensitive (SS), heterozygote (RS) and homozygote resistant (RR). Three salmon infested with Ls F were treated for 30 minutes with 0.1 mgL^-1^ azamethiphos in a bath treatment. All detached salmon lice were removed during the exposure and the following 2.5 hours. Detached parasites were also removed 24 hours later. Two parasites were excluded from the analysis as they attached to the tank wall after detaching from the fish. The rest of the parasites were picked off the fish 8 days post treatment. These salmon lice were regarded as alive, while the detached were regarded as dead. All salmon lice were genotyped by PatoGen AS in Ålesund, Norway using a TaqMan assay. The upper dotted line is the RR group, the solid line is the RS group, while the lower broken line is the SS group. One of the RS parasites died between 200 minutes and 24 hours after start of exposure, but the exact time is unknown. In this plot the time of death is set to 250 minutes. The cut-off limit is set to 300 minutes.

The Kaplan-Meier survival analysis of the SS genotype demonstrated that the median survival time was 25 minutes (95% CI: 15–30 min). A Wilcoxon test showed highly significant differences between the groups (χ^2^ = 64.7, DF = 2, p< 0.0001). The survival plot is displayed in Fig 6.

### Enzyme inhibition assay

The degree of enzymatic inhibition was assessed both *in vitro* (with propoxur added to lice homogenates) and *in vivo* (with azamethiphos exposure of live salmon lice). One azamethiphos-susceptible and one-resistant strain of parasites were used, representing the genotypes SS and RR, respectively.

**Fig 7:**
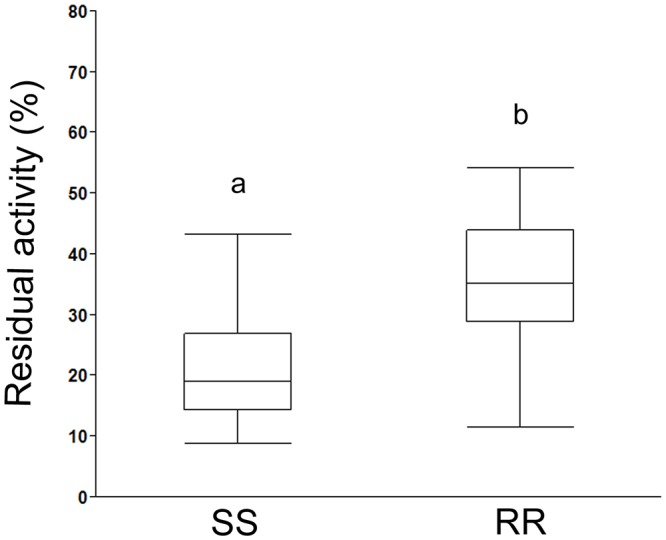
Residual enzyme activity after *in vitro* inhibition. The relative residual AChE activity with or without propoxur in susceptible (SS, n = 20) and resistant (RR, n = 21) lice is displayed. A statistically significant effect on residual AChE activity was found between the two groups, indicating a protective effect of the *Phe362Tyr* mutation against propoxur inhibition in homogenates (p<0.0001, ANOVA). The box plots indicate the group median, 75% and 25% quantiles, and whiskers (JMP, SAS Institute). Different letters indicate a statistically significant difference.

In the *in vitro* assay, residual activity was calculated in homogenates with propoxur added to the wells and normalized to the activity in the non-inhibited fraction. A statistically significant difference was found in residual activity between the two genotypes SS and RR (p<0.0001). The arithmetic means of the residual activities were 21.1% and 37.3% with confidence intervals (95%) of [16.1; 26.2] and [32.4; 42.2] for SS and RR, respectively (Fig 7).

**Fig 8:**
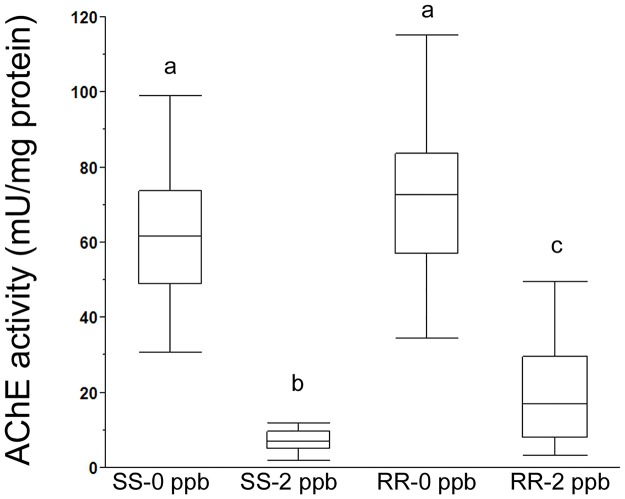
Residual enzyme activity after *in vivo* inhibition. AChE activity (mU/mg protein) in susceptible (SS) and resistant (RR) lice after treatment with 0 μgL^-1^ (control) or 2 μgL^-1^ azamethiphos for 24 hours. No difference was observed between the control groups. A statistically significant decrease in the residual activity after 2 μgL^-1^ azamethiphos treatment was found in both strains (p<0.0001, Steel-Dwass method). In addition, there was a statistically significant difference between the two treated groups (SS-2 μgL^-1^ and RR-2 μgL^-1^; p = 0.019, Steel-Dwass method) indicating a protective effect of the *Phe362Tyr* mutation against azamethiphos bath treatment). The box plots indicate the group median, 75% and 25% quantiles, and whiskers (JMP, SAS institute). Different letters indicate a statistically significant difference.

Inhibition with azamethiphos was done on live lice (*in vivo*); hence one louse could only belong to one of the treatment groups. In contrast to the *in vitro* experiments, residual activity could therefore not be normalized. Instead, the absolute values of enzyme activity were used to compare the four groups (Fig 8). Azamethiphos, 2 μgL^-1^, inhibited AChE activity in both the susceptible strain (SS; p<0.0001) and the resistant strain (RR; p<0.0001). In the susceptible strain (SS) the median activities were 61.6 mU/mg protein (0 μgL^-1^) and 7.0 mU/mg protein (2 μgL^-1^) and in the resistant strain (RR) the median activities were 72.7 mU/mg protein (0 μgL^-1^) and 16.9 mU/mg protein (2 μgL^-1^), respectively. No statistically significant difference was found between SS and RR control groups (not exposed to azamethiphos). After exposure to 2 μgL^-1^ azamethiphos, there was a significant difference in absolute residual activity between the two genotypes (p = 0.019), indicating that the *L*. *salmonis* mutation *Phe362Tyr* is involved in the protection against azamethiphos. All the lice in the SS-2 μgL^-1^ group were immobilized when sampled. In the RR-2 μgL^-1^ group, the behavior was not notably different from the control group (RR-0 μgL^-1^), as only 6.8% and 4.7% were immobilized at the end of the observation period, respectively. The AChE activities in all of the samples from the SS group were below the median value in the RR group.

## Discussion

Decreased sensitivity for various chemotherapeutics has become a major issue in controlling the sea lice problem worldwide with Norway being no exception [5]. Azamethiphos has been one of the most commonly used chemical treatment agents against sea lice in Norway for decades [3]. However, the development of resistance over the years, attributed to its overuse, has affected the fish farm industry a lot. Unfortunately, no tool is yet available to identify resistance because of the lack of knowledge about the molecular mechanisms involved in resistance.

As per the existing literature, resistance towards azamethiphos is mostly associated with mutations in AChE genes in various arthropods [7]. Among these, point mutations are the most commonly found mutations [7]. Around 70 different mutations have been reported in AChE genes to be associated with decreased sensitivity against azamethiphos in various species [7]. Majority of these missense mutations have been found in or around the active gorge site of the enzyme, making it a hot spot for mutations [7].

In the present study, we investigated the molecular mechanisms of azamethiphos resistance in *L*. *salmonis*. Based on our results we could substantially state that *L*. *salmonis ace1a* is the primary target for azamethiphos and that the *Phe362Tyr* replacement (corresponding to *Phe331* in *T*. *californica*) is primarily responsible for conferring reduced sensitivity in *L*. *salmonis* against azamethiphos.

*Phe362* position in *L*. *salmonis* is homologous to *Phe331* position in *T*. *californica* and is located in the acyl pocket neighboring the active centre in the active site gorge (Fig 4) [17]. The acyl pocket is responsible for ligand specificity via two properties. The first property is related to the formation of the enzyme-substrate intermediate complex. The acyl pocket is located at the bottom of the active site and surrounded by side chains of hydrophobic aromatic residues. Because of its location and surroundings, acyl pocket attracts and orientates the acyl group of the substrate and inhibitors through its hydrophobicity during the catalytic reaction [17, 18].

The second property of the acyl pocket derives from its electrostatic field. In *T*. *californica*, the pocket forming the side chain of *Phe331* attracts the catalytic *His440* by cation-*π* interaction. *Phe331* is considered to arrange the catalytic histidine so that proper conformational change of the histidine can occur in the hydrolyzing step. Mutagenesis studies followed by computer simulation demonstrated that the orientation of *His447* in human AChE (corresponds to *His440* in *T*. *californica*) is changed by substitution from the wild type *Phe338* (*Phe331* in *T*. *californica*) to aliphatic residues [19, 20]. However, whether a substitution with Tyr at this position affects the cation- *π* interaction is not known. Nevertheless, this substitution in the *L*. *salmonis* AChE1a is considered to affect the inhibitor enzyme interaction either by changes in inhibitor affinity or interaction with the catalytic histidine, or both.

In the present study, the 3D model of the AChE1a in *L*. *salmonis* revealed that the *Phe362Tyr* substitution makes the acyl pocket smaller and more polar (Fig 4), which could alter the accessibility of azamethiphos to the site. The docking of azamethiphos to the enzyme also suggested that both *362Tyr* and *Tyr152* formed H-bonds with azamethiphos in the mutated enzyme, thereby interfering with the capability of azamethiphos to bind to serine in position 230 (*Ser200* in *T*. *californica*). In addition, the mutation screening revealed a significantly higher frequency of *362Tyr* in resistant samples compared to the sensitive samples (Table 3). Moreover, all the survivors of azamethiphos treatment of the Ls H strain, (Ls H-s) carried *362Tyr* mutant allele (Table 3); increasing the frequency of *362Tyr* from 56% to 100% (92% samples with one mutant allele (RS) and 8% samples with both the mutant alleles (RR). This clearly indicates the importance of *362Tyr* in the survival of sea lice under azamethiphos exposure.

The association between the mutation *Phe362Tyr* and salmon lice resistance against OPs was further supported by enzymatic assays of AChE activity using the two inhibitors (propoxur and azamethiphos) in one *in vitro* and one *in vivo* assay, respectively. Propoxur inhibited AChE activity at a significantly lower degree in resistant *L*. *salmonis* with the *Phe362Tyr* mutation (RR) compared to salmon lice without the mutation (SS). This is in accordance with a standardized assay developed by the World Health Organization (WHO) to assess insecticide resistance in mosquitoes [15] although the cut-off for classifying resistance in mosquitos does not apply to salmon lice. An important reason for this is the presence of two AChEs in salmon lice [8]. The assay was done on homogenate of whole louse, thus both AChE1a and AChE1b contributed to the total enzymatic activity measured. However, the relative contribution between the two AChEs and their significance for the salmon lice to survive has not yet been clarified. In mosquitos only one AChE has been characterized. Therefore, a resistance-related mutation would be significantly more effective in preventing propoxur inhibition, as shown in *Anopheles subpictus* [21], *Anopheles maculipennis* [22] and *Culex quinquefasciatus* [23]. The *in vitro* results suggest a substantial contribution of AChE1b to the total AChE-activity in *L*. *salmonis*. As no resistance-associated mutations were found in AChE1b, this enzyme was assumed to be fully inhibited by propoxur in both the SS and the RR group. Thus, it is the AChE1a residual activity in *362Tyr* samples compared to *Phe362* samples that renders the parasite capable of surviving an azamethiphos treatment. This was examined in the *in vivo experiment* and ties the link between the biochemical effect alone and the survival after azamethiphos treatment. The results confirm that there was a difference in residual activity also after exposure of live parasites to azamethiphos. The somewhat greater dispersion of the data points in the RR group suggest that the relative contribution of AChE1a and AChE1b can vary between individuals. Thus, a cut-off limit for the total AChE activity cannot be used as an indicator for the *Phe362Tyr* mutation until more knowledge on the different contribution of the two proteins, both quantitatively and qualitatively, has been generated.

The low frequency of *362Tyr* in the samples without azametiphos treatment (Ls G, 4%) could be explained by the theory suggested by Shi *et al*. (2004), which states that even though there is a fitness cost associated with mutations in AChE, conferring resistance towards OP, the alleles might still survive without selection pressure [24]. The frequency of the mutant allele in the natural population (without treatment pressure) depends on the alteration caused by mutant allele on the protein. The point mutations cause a low level of alteration in the protein, which is the main driving force responsible for the maintenance of resistant alleles in natural populations [24]. This theory is supported by the fact that most of the mutations reported in AChE are point mutations [7]. Further, the presence of *362Tyr* in the samples from 1998 is another evidence in support of the theory, as this ascertains the presence of mutant (*362Tyr*) allele in the salmon lice population, without selection pressure, for eight (2000–2007) years [25, 26].

Around 70 different missense mutations have been reported to be associated with OP resistance in *ace* genes from other species [7]. Interestingly, none of these missense mutations were found in the *ace1a* gene in our resistant samples, neither in the samples from 1998, nor in later samples. This observation suggests a single origin of *Phe362Tyr* mutation, which dispersed intensively due to the immense selection pressure caused by repeated OP treatments in salmon farms. As the first cases of OP resistance in salmon lice were reported in 1991 [4], it is most likely that the mutation was originated at that time.

The observations of the present study very well supported the theory by Shi *et al*. (2004) [24]. As the mutant allele could affect the fitness of the salmon lice (without azamethiphos treatment), the frequency of samples carrying *362Tyr* goes down (4%) in the natural population (Ls G). However, the mutant allele (*362Tyr)* persists in the natural population along with the wild type allele (*Phe362*) as the fitness cost is limited. After treatment the frequency of *362Tyr* shoots up (100% in Ls H-s, Table 3; of which 92% carried one mutant allele and the remaining were homozygous for *362Tyr*) i.e. only the carriers of the mutant allele (*362Tyr)* survived the treatment.

The mutation screening experiment was further validated by the small scale treatment experiment (Fig 6), which showed that all the samples without *Phe362Tyr* substitution (SS) died within 2 hours of the treatment whereas no mortality was seen among the homozygous samples (RR) for *362Tyr* substitution. 44% of the samples that carried only one mutated allele (RS) died. This observation again indicated that *362Tyr* plays a vital role in the survival of salmon lice under azamethiphos treatment.

*Phe331* (numbering from *T*. *californica*) is highly conserved among AChE1 and AChE2 from various species as shown in Fig 3. The high conservation of *Phe331* and its location in AChE at an important site, signals its potential significance in the protein function and in turn the survival of organism (Fig 3 and Fig 4).

Together, all the observations of the present study, clearly point towards a strong association of the *Phe362Tyr* substitution in *L*. *salmonis* with decreased sensitivity of sea lice towards azamethiphos.

To the best of our knowledge, *Phe362Tyr* mutation (codon 331 in *T*. *californica*) has not been reported earlier. However, there are reports about other resistance-associated point mutations at the same amino acid position. For example, substitution of Ser with Phe in this position in AChE2 of *Myzus persicae* was found to be associated with insensitivity towards pirimicarb [27], and towards pirimicarb and omethoate in AChE1 of *Ashbya gossypii* (*Ser431Phe* in this species) [28]. Alon *et al*. (2008) have reported *Phe392Trp* (*Phe331* in *T*. *californica*) substitution in AChE1 of *Bemisia tabaci* [29]. A similar mutation, *Phe455Trp* (*Phe331* in *T*. *californica*), was reported in AChE2 of *Culex tritaeniorhynchus*, in association with extreme insecticide insensitivity (30-fold) and was considered to be solely responsible for the insecticide—resistance of AChE in these mosquitoes [30, 31]. Expression of this mutation in AChE1 from *C*. *tritaeniorhynchus* in a baculovirus-Sf9 cell system and subsequent treatment of the expressed proteins with OP and carbamate inhibitors revealed extremely reduced sensitivity to OP compounds [31]. Anazawa *et al*. (2003) observed a 140 fold decrease in sensitivity towards the OP dichlorvos in *Tetranychus urtricae* with a change from Phe to Cys at the same position [32]. Similarly, Kwon *et al*. (2012) found a 99 fold decrease in sensitivity to monocrotophos and a significant decrease in catalytic efficiency of the enzyme in *T*. *urticae* carrying a *Phe439Trp* (*Phe331Trp* in *T*. *californica*) mutation [33].

Mutagenesis studies with human AChE also demonstrated that *Phe338* (*Phe331* in *T*. *californica*) to Ala replacement conferred a 2-fold decrease in edrophonium-senstivity [34].

Various studies involving the mutagenized and naturally occurring substitutions in insect AChEs have also inferred the importance of positions homologous to the *T*. *californica Phe331* position, for reduced sensitivity towards AChE-inhibiting insecticides. For example, an *in vitro* mutagenesis study carried out with *Drosophila melanogaster ace2*, demonstrated that substitutions of *Phe371* (homologous position in *D*. *melanogaster ace2* to *Phe331* in *T*. *californica*) to Ala, Gly, Ile and Tyr resulted in 10–100 fold decrease in carbamate sensitivity. Interestingly, a 100 fold decrease in carbaryl, malaoxon and paraoxon sensitivity with *Phe371Tyr* (*Phe331Tyr* in *T*. *californica*) substitution was observed by site-directed mutagenesis [35]. This engineered mutation is homologous to the natural mutation described in the current study and further strengthens the importance of *Phe331* in sensitivity towards OPs, as well as the significance of the described *Phe362Tyr* mutation in *L*. *salmonis*.

In conclusion, four lines of evidence for the significance of the *Phe362Tyr* (*L*. *salmonis*) mutation in relation to salmon lice resistance towards azamethiphos are presented here. Firstly, the significantly high frequency of *362Tyr* in *L*. *salmonis* samples resistant to azamethiphos indicated a clear association of *Phe362Tyr* with reduced sensitivity towards azamethiphos (Table 3). Secondly, the 3D modelling suggested that *362Tyr* could affect the access and binding of azamethiphos at the active site (Fig 4). Thirdly, the treatment trial for genetic characterization with azamethiphos showed 0% mortality in samples with both the mutated (*362Tyr*) alleles (Table 3). And finally the enzymatic assay revealed a significantly higher residual activity in resistant (RR) versus the sensitive (SS) samples (Fig 7 and Fig 8) both *in vitro* and *in vivo*. Taken together, all these observations provide a strong argument in favor of *Phe362Tyr* mutation being the culprit behind azamethiphos resistance in *L*. *salmonis*.

## Supporting Information

S1 File PDB-file of the wild-type AChE1a in *Lepeophtheirus salmonis*. (TXT)

S2 File PDB-file of the mutated AChE1a in *Lepeophtheirus salmonis*. (TXT)

S1 Table Frequency of the Ile433Thr change in AChE1b in *Lepeophtheirus salmonis*. (DOCX)

## References

[1] Aldridge WN. Some properties of specific cholinesterase with particular reference to the mechanism of inhibition by diethyl p-nitrophenyl thiophosphate (E 605) and analogues. Biochem J. 1950;46: 451–460. 15420172 10.1042/bj0460451 PMC1275447

[2] Fournier D, Mutero A. Modification of acetylcholinesterase as a mechanism of resistance to insecticides. Comp Biochem Phys C. 1994;108: 19–31.

[3] Torrissen O, Jones S, Asche F, Guttormsen A, Skilbrei OT, Nilsen F et al Salmon lice—impact on wild salmonids and salmon aquaculture. J Fish Dis. 213; 36: 171–194. 23311858 10.1111/jfd.12061 PMC3675643

[4] Denholm I, Devine GJ, Horsberg TE, Sevatdal S, Fallang A, Nolan DV et al Analysis and management of resistance to chemotherapeutants in salmon lice, *Lepeophtheirus salmonis* (Copepoda: Caligidae). Pest Manag Sci. 2002;58: 528–536. 12138619 10.1002/ps.482

[5] Grøntvedt RN, Jansen PA, Horsberg TA, Helgesen K, Tarpai A. The surveillance programme for resistance to chemotherapeutants in *L*. *salmonis* in Norway. Surveillance programmes for terrestrial and aquatic animals in Norway Annual report 2013. Oslo: Norwegian Veterinary Institute 2014.

[6] Riaz MA, Chandor-Proust A, Dauphin-Villemant C, Poupardin R, Jones CM, Strode C et al Molecular mechanisms associated with increased tolerance to the neonicotinoid insecticide imidacloprid in the dengue vector *Aedes aegypti* . Aquat Toxicol. 2013;126: 326–337. 10.1016/j.aquatox.2012.09.010 23058251

[7] Hotelier T, Nègre V, Marchot P, Chatonnet A. Insecticide resistance through mutations in cholinesterases or carboxylesterases: data mining in the ESTHER database. J Pestic Sci. 2010;35: 315–320.

[8] Kaur K, Bakke MJ, Nilsen F, Horsberg TE. Identification and molecular characterization of two acetylcholinesterases from the salmon louse, *Lepeophtheirus salmonis* , PLOS ONE. 2015. In press 10.1371/journal.pone.0125362 PMC4418574 25938836

[9] Hamre LA, Glover KA, Nilsen F. Establishment and characterisation of salmon louse (*Lepeophtheirus salmonis* (Krøyer 1837)) laboratory strains. Parasitol Int. 2009;58: 451–460. 10.1016/j.parint.2009.08.009 19732850

[10] Helgesen KO, Horsberg TE. Single-dose field bioassay for sensitivity testing in sea lice, *Lepeophtheirus salmonis*: development of a rapid diagnostic tool. J Fish Dis. 2013;36:261–272. 10.1111/jfd.12053 23298397

[11] Reed GH, Kent JO, Wittwer CT. High-resolution DNA melting analysis for simple and efficient molecular diagnostics. Pharmacogenomics 2007;8: 597–608. 17559349 10.2217/14622416.8.6.597

[12] Thompson JD, Higgins DG, Gibson TJ. CLUSTAL W: improving the sensitivity of progressive multiple sequence alignment through sequence weighting, position-specific gap penalties and weight matrix choice. Nucleic Acids Res. 1994;22: 4673–4680. 7984417 10.1093/nar/22.22.4673 PMC308517

[13] Arnold K, Bordoli L, Kopp J, Schwede T. The SWISS-MODEL Workspace: A web-based environment for protein structure homology modelling. Bioinformatics 2006;22:195–201. 16301204 10.1093/bioinformatics/bti770

[14] Harel M, Kryger G, Rosenberry TL, Mallender WD, Lewis T, Fletcher RJ et al Three-dimensional structures of *Drosophila melanogaster* acetylcholinesterase and of its complexes with two potent inhibitors. Protein Sci. 2000;9:1063–1072. 10892800 10.1110/ps.9.6.1063 PMC2144661

[15] WHO (World Health Organization). Techniques to detect insecticide resistance mechanisms (field and laboratory manual). WHO/ CDS/CPC/MAL/98.6. 1998

[16] Ellman GL, Courtney KD, Andres V Jr, Feather-Stone RM. A new and rapid colorimetric determination of acetylcholinesterase activity. Biochem Pharmacol. 1961;7: 88–95. 13726518 10.1016/0006-2952(61)90145-9

[17] Hosea NA, Berman HA, Taylor P (1995) Specificity and orientation of trigonal carboxyl esters and tetrahedral alkylphosphonyl esters in cholinesterases. Biochemistry 1995;34:11528–11536. 7547883 10.1021/bi00036a028

[18] Ordentlich A, Barak D, Kronman C, Benschop HP, De Jong LP, Ariel N et al Exploring the active center of human acetylcholinesterase with stereomers of an organophosphorus inhibitor with two chiral centers. Biochemistry 1999;38: 3055–3066. 10074358 10.1021/bi982261f

[19] Shafferman A, Ordentlich A, Barak D, Stein D, Ariel N, Velan B. Aging of phosphylated human acetylcholinesterase: catalytic processes mediated by aromatic and polar residues of the active centre. Biochem J. 1996;318: 833–840. 8836126 10.1042/bj3180833 PMC1217693

[20] Bar-On P, Millard CB, Harel M, Dvir H, Enz A, Sussman JL et al Kinetic and structural studies on the interaction of cholinesterases with the anti-Alzheimer drug rivastigmine. Biochemistry 2002;41: 3555–3564. 11888271 10.1021/bi020016x

[21] Surendran SN, Jude PJ, Weerarathne TC, Parakrama Karunaratne SHP, Ramasamy R. Variations in susceptibility to common insecticides and resistance mechanisms among morphologically identified sibling species of the malaria vector *Anopheles subpictus* in Sri Lanka. Parasit Vectors 2012;5:34 10.1186/1756-3305-5-34 22325737 PMC3317438

[22] Muhammet MA. Malathion and Propoxur Resistance in Turkish Populations of the *Anopheles maculipennis* Meigen (Diptera: Culicidae) and Relation to the Insensitive Acetylcholinesterase. Turkiye Parazitol Derg. 2014;38:111–115. 10.5152/tpd.2014.3388 25016118

[23] Low VL, Chen CD, Lee HL, Tan TK, Chen CF, Leong CS et al Enzymatic characterization of insecticide resistance mechanisms in field populations of Malaysian *Culex quinquefasciatus* Say (Diptera: Culicidae). PLOS ONE 2013;8:e79928 10.1371/journal.pone.0079928 24278220 PMC3836847

[24] Shi MA, Lougarre A, Alies C, Frémaux I, Tang ZH, Stojan J et al (2004) Acetylcholinesterase alterations reveal the fitness cost of mutations conferring insecticide resistance. BMC Evol Biol. 2004;4: 5 15018650 10.1186/1471-2148-4-5 PMC362868

[25] Grave K, Horsberg TE, Lunestad BT, Litleskare I. Consumption of drugs for sea lice infestations in Norwegian fish farms: methods for assessment of treatment patterns and treatment rate. Dis Aquat Organ. 2004;60: 123–131. 15460856 10.3354/dao060123

[26] Helgesen KO, Bravo S, Sevatdal S, Mendoza J, Horsberg TE.Deltamethrin resistance in the sea louse *Caligus rogercresseyi* (Boxhall and Bravo) in Chile: bioassay results and usage data for antiparasitic agents with references to Norwegian conditions. J Fish Dis 2014;37: 877–890. 10.1111/jfd.12223 24697556

[27] Nabeshima T, Kozaki T, Tomita T, Kono Y. An amino acid substitution on the second acetylcholinesterase in the pirimicarb-resistant strains of the peach potato aphid, *Myzus persicae* . Biochem Biophys Res Commun. 2003;307:15–22. 12849975 10.1016/s0006-291x(03)01101-x

[28] Benting J, Nauen R. Biochemical evidence that an S431F mutation in acetylcholinesterase-1 of *Aphis gossypii* mediates resistance to pirimicarb and omethoate. Pest Manag Sci. 2004;60:1051–1055. 15532677 10.1002/ps.971

[29] Alon M, Alon F, Nauen R, Morin S. Organophosphates' resistance in the B-biotype of *Bemisia tabaci* (Hemiptera: Aleyrodidae) is associated with a point mutation in an ace1-type acetylcholinesterase and overexpression of carboxylesterase. Insect Biochem Mol Biol. 2008;38: 940–949. 10.1016/j.ibmb.2008.07.007 18721883

[30] Nabeshima T, Mori A, Kozaki T, Iwata Y, Hidoh O, Harada S et al An amino acid substitution attributable to insecticide-insensitivity of acetylcholinesterase in a Japanese encephalitis vector mosquito, *Culex tritaeniorhynchus* . Biochem Biophys Res Commun 2004;313:794–801. 14697262 10.1016/j.bbrc.2003.11.141

[31] Oh S-H, Kozaki T, Mizuno H, Tomita T, Kono Y (2006) Expression of Ace-paralogous acetylcholinesterase of *Culex tritaeniorhynchus* with an amino acid substitution conferring insecticide insensitivity in baculovirus-insect cell system. Pestic Biochem Phys 2006;85: 46–51.

[32] Anazawa Y, Tomita T, Aiki Y, Kozaki T, Kono Y. Sequence of a cDNA encoding acetylcholinesterase from susceptible and resistant two-spotted spider mite, *Tetranychus urticae* . Insect Biochem Mol Biol. 2003;33: 509–514. 12706630 10.1016/s0965-1748(03)00025-0

[33] Kwon DH, Choi JY, Je YH, Lee SH. The overexpression of acetylcholinesterase compensates for the reduced catalytic activity caused by resistance-conferring mutations in *Tetranychus urticae* . Insect Biochem Mol Biol. 2012;42: 212–219. 10.1016/j.ibmb.2011.12.003 22198354

[34] Shafferman A, Velan B, Ordentlich A, Kronman C, Grosfeld H, Leitner M et al Substrate inhibition of acetylcholinesterase: residues affecting signal transduction from the surface to the catalytic center. EMBO J. 1992;11: 3561–3568. 1396557 10.1002/j.1460-2075.1992.tb05439.x PMC556814

[35] Boublik Y, Saint-Aguet P, Lougarre A, Arnaud M, Villatte F, Estrada-Mondaca S et al (2002) Acetylcholinesterase engineering for detection of insecticide residues. Protein Eng. 2002;15: 43–50. 11842237 10.1093/protein/15.1.43

