# Supplementary material for: Mechanism behind Resistance against the Organophosphate Azamethiphos in Salmon Lice (Lepeophtheirus salmonis)
Source: PLoS One. 2015 Apr 20;10(4):e0124220. doi: 10.1371/journal.pone.0124220 (PMC4403986; doi:10.1371/journal.pone.0124220)
Supplement: S1 Table — (DOCX) [file pone.0124220.s003.docx]

**Table S1**. Frequency of the Ile->Thr change in codon 433 of *ace1b* (*L. salmonis*)

| **Strain** | **Sensitivity** | **Ile433Thr** |
| --- | --- | --- |
| Ls A | Sensitive | 80 % |
| Ls G | Sensitive | 86 % |
| Ls B | Reduced sensitivity | 88 % |
| Ls H | Resistant | 98 % |
